# Evaluatio*N* of *A*pi*X*aban in str*O*ke and systemic embolism prevention in patients with non‐valvular atrial fibrillation in clinical practice *S*etting in France, rationale and design of the NAXOS: SNIIRAM study

**DOI:** 10.1002/clc.23231

**Published:** 2019-07-17

**Authors:** Fabien Picard, Eric Van Ganse, Gregory Ducrocq, Nicolas Danchin, Bruno Falissard, Olivier Hanon, Manon Belhassen, Marine Ginoux, Cinira Lefevre, François‐Emery Cotte, Isabelle Mahé, Philippe G. Steg

**Affiliations:** ^1^ Department of Cardiology Cochin Hospital, AP‐HP Paris France; ^2^ Université Paris Descartes Paris France; ^3^ FACT (French Alliance for Cardiovascular Trials) Paris France; ^4^ PharmacoEpidemiology Lyon (PELyon), EA 7425 HESPER Health Services and Performance Research Claude‐Bernard University Lyon France; ^5^ Respiratory Medicine Croix‐Rousse Hospital Lyon France; ^6^ Département de cardiologie, Hôpital Bichat Assistance Publique‐Hôpitaux de Paris, DHU FIRE, INSERM 1148, Université de Paris Paris France; ^7^ Department of Cardiology Georges Pompidou European Hospital, AP‐HP Paris France; ^8^ U669 – Hôpital Cochin Maison des adolescents, AP‐HP Paris France; ^9^ Hôpital Broca 54‐56 Pascal, 75013 Paris France; ^10^ Université Paris Descartes Sorbonne Paris Cité, Equipe d'Accueil 4468 Paris France; ^11^ PharmacoEpidemiology Lyon (PELyon) Lyon France; ^12^ Bristol‐Myers Squibb Market Access Rueil‐Malmaison France; ^13^ Bristol‐Myers Squibb Health Economics & Outcomes Research Rueil‐Malmaison France; ^14^ Department of Internal Medicine Louis‐Mourier Hospital, Universite Paris 7, Inserm UMR_S1140, AP‐HP Colombes France; ^15^ National Heart and Lung Institute Royal Brompton Hospital, Imperial College London UK

**Keywords:** apixaban, major bleeding, non‐valvular atrial fibrillation, non‐vitamin K antagonist oral anticoagulants, systemic embolism, vitamin‐K antagonists

## Abstract

Non‐vitamin K antagonists oral anticoagulants (NOACs) have recently challenged vitamin‐K antagonists (VKAs) for stroke and systemic embolism prophylaxis in patients with non‐valvular atrial fibrillation (NVAF). Nevertheless, little information is available in routine clinical practice for France. The aim of this study is to describe the effectiveness and safety of apixaban, rivaroxaban, dabigatran or VKAs in routine clinical practice in adult NVAF patients for the prevention of stroke and systemic embolism in France. The NAXOS study is a nationwide observational retrospective cohort generated from the French national healthcare insurance database (SNIIRAM—a comprehensive in‐ and outpatient healthcare consumption database), consisting of eight distinct sub‐cohorts of anticoagulant‐naive or anticoagulant‐experienced patients diagnosed with NVAF, newly initiated with either NOACs (dabigatran, rivaroxaban or apixaban) or VKAs. Patients will be included if initiating a new anticoagulant treatment for AF during the study period from 1 January 2014 to 31 December 2016. Primary effectiveness outcome will be the incidence of stroke or systemic thromboembolic events; primary safety outcome will be the incidence of major bleeding during the exposure period. The NAXOS study will provide routine clinical practice data on the effectiveness and safety profiles of apixaban vs other NOACs and VKAs in the prevention of stroke and systemic embolism in adult patients with NVAF in clinical practice conditions in France.

**Abbreviations**
AF: atrial fibrillation
CMA: Continuous Measure of Medication Acquisition
CNIL: French data protection commission, “*Commission Nationale Informatique et Libertés*”
ICD: International Classification of Diseases
IDS: Institute of health data, “*Institut des Données de Santé*”
LTD: long‐term disease, “*Affection Longue Durée*”
NOACs: non‐vitamin K antagonist oral anticoagulants
NVAF: non‐valvular atrial fibrillation
PMSI: French Hospital Discharge database
SNIIRAM: French National Health Insurance Information System
VKAs: vitamin‐K antagonists

## 1 INTRODUCTION

Atrial fibrillation (AF) can lead to significant mortality, morbidity, and cost.^1^, ^2^ Long‐term prophylaxis with anticoagulation therapy is recommended to prevent stroke and systemic embolization in patients with AF presenting an independent risk factor for stroke.^3^ Four non vitamin‐K antagonists oral anticoagulants (NOACs) are currently available: the direct factor Xa inhibitors (rivaroxaban, apixaban, and edoxaban) and the direct factor IIa inhibitor (dabigatran). These four NOACs have demonstrated, in randomized clinical trials, a consistent favorable risk‐benefit profile across a wide range of patients with reductions in stroke or systemic embolism, intracranial hemorrhage, and mortality but increased gastrointestinal bleeds when compared with warfarin.^4^, ^5^, ^6^, ^7^, ^8^

Clinical practice data regarding routine use of the different NOACs largely mirrors the results of clinical trials.^9^, ^10^, ^11^, ^12^, ^13^ In addition, a recent study using the French medico‐administrative databases (SNIIRAM and PMSI), including patients with NVAF who initiated dabigatran or rivaroxaban was previously reported but did not include data on apixaban.^14^ The French Health Authority (HAS) Transparency Commission granted positive appraisal in June 2013 to the reimbursement of apixaban in the indication of stroke prevention and systemic embolism in patients with NVAF, with one or more risk factors, such as prior stroke or transient ischemic attack, age ≥ 75 years, hypertension, diabetes mellitus, and symptomatic heart failure (NYHA ≥ class II). However, while recommending the reimbursement of apixaban in this indication, HAS requested clinical practice data documenting the therapeutic benefit of apixaban under actual conditions of use, compared with the standard oral anticoagulant treatment recommended in France (vitamin‐K antagonists, dabigatran, and rivaroxaban). Indeed, limited clinical practice data are available on the characteristics, clinical management, and outcomes of patients treated with apixaban in France. The availability of warfarin and three NOACs in France allows opportunities for comparative analyses, particularly on the effectiveness and the safety of these drugs when used outside the controlled setting of clinical trials. Nevertheless, as edoxaban was only recently introduced to the market, and not yet available in France, this analysis will only focus on VKAs, dabigatran, rivaroxaban, and apixaban.

The NAXOS study is a nationwide observational retrospective cohort generated from the French National healthcare claims database (SNIIRAM) from which eight distinct sub‐cohorts of anticoagulant‐naive and anticoagulant‐experienced patients diagnosed with NVAF will be identified. The aim of this study is to estimate in a clinical practice setting in France the risk of stroke and systemic thromboembolism, and major bleeding events in NVAF patients treated with various anticoagulants; to describe baseline characteristics; and to compare outcomes between patients initiated by apixaban vs patients initiated by each of the other anticoagulants.

## 2 STUDY OBJECTIVES

Primary objectives:

- *Effectiveness objective*: To estimate the risk of stroke or systemic thromboembolic events according to the newly initiated anticoagulant treatment (corresponding to the first anticoagulant treatment recorded in the database during the study period), in NVAF patients.
- *Safety objective*: To estimate the risk of major bleeding according to the newly initiated anticoagulant treatment, in NVAF patients.

Secondary objectives: In NVAF patients, and according to the OAC treatment, the study intended:

- To describe demographic and clinical characteristics.
- To estimate the risk of occurrence of a composite morbidity criterion of stroke, systemic embolism, and major bleeding.
- To estimate the risk of all‐cause mortality.
- To describe treatment patterns at anticoagulant initiation and over time.
- To estimate the healthcare resource utilization.
- To estimate the off‐label use of apixaban in the French valvular AF population initiating apixaban.
- In OAC‐naive patients only: To compare baseline characteristics, outcomes (stroke and systemic thromboembolic events, major bleeding, and all‐cause mortality), and healthcare resource utilization rates between patients initiated with apixaban vs patients initiated with each of the other anticoagulant treatments in anticoagulant‐naive patients.

## 3 METHODS

### 3.1 Data source and study population

**Figure 1:**
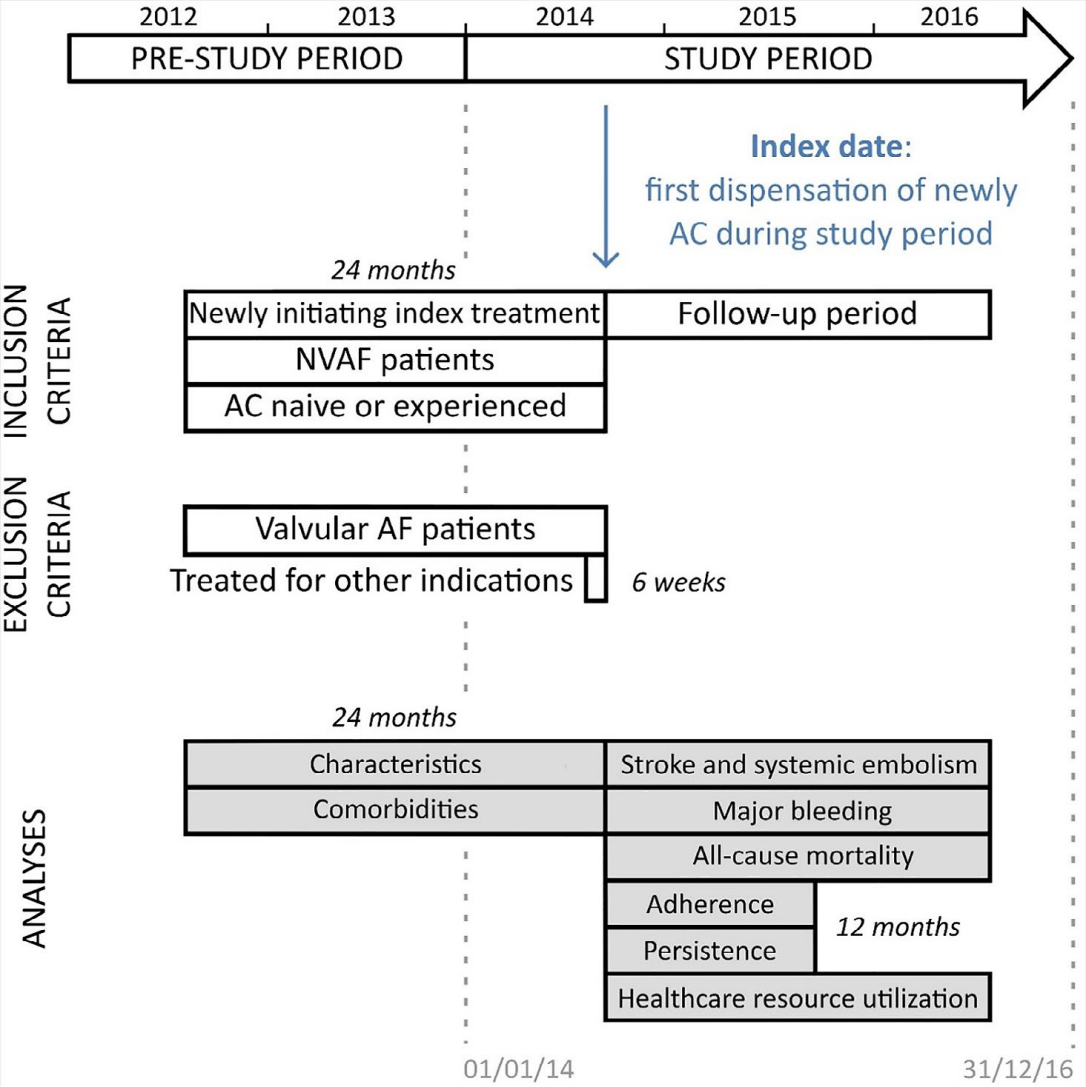
Study design. AC, anticoagulant; AF, atrial fibrillation; NVAF, non‐valvular atrial fibrillation

The NAXOS study is a French nationwide, observational retrospective cohort of NVAF patients newly initiated with either apixaban, dabigatran, rivaroxaban or VKAs, from which eight distinct sub‐cohorts (Figure 1) of anticoagulant‐naive patients and anticoagulant‐experienced patients (patients treated by anticoagulant before the inclusion date) will be identified.

Using the French National Health Insurance System (SNIIRAM) database, patients were identified and included in the study if they initiated a new anticoagulant treatment from 1 January 2014 to 31 December 31 2016. The study will be led by a project team, a coordinating center and a scientific board which is detailed in the Supporting Information Appendix 1.

Patients will be followed from the date of their first OAC prescription until the end of follow‐up, which is defined by the occurrence of a following event, whichever comes first:

1. *Switch to another anticoagulant treatment*: defined as dispensation of another anticoagulant molecule recorded after initiation of the studied anticoagulant treatment. Date of switch (and end of follow‐up) will be the date of the first dispensation of the other anticoagulant molecule.
2. *Discontinuation*: a patient who remains more than 30 days after the coverage by the last dispensation of studied anticoagulant treatment without refilling it will be considered as stopping this treatment. If a hospitalization occurs within this period, the length of the hospital stay will be deducted from the number of days without refilling of the treatment. For patients treated with VKAs, International Normalized Ratio testing realized during a hospitalization will be counted as a VKA dispensation. International Normalized Ratio testing will be used as a proxy of a VKA prescription only to extend VKA treatment exposure, but it will not be used as an index date. OACs drug coverages will be derived based on the recommended dosage, and the coverage of VKA treatment exposure will be defined as 35 days (ie, the median coverage time in days of VKA treatments, based on prior assessment on claims database). The median number of coverage days will also be calculated in the VKA patient population after data extraction.
3. *Last patient's health record*: defined by the last care (ie, consultations, dispensations, medical procedures…) recorded in the database before a period of 6 months without any reimbursed care. This includes situations like emigration and geriatric homes entry.
4. *Patient's death*.
5. *End of study period*.

#### 3.1.1 Data source

The data source used for this study was the SNIIRAM database, which is the French national healthcare insurance system database with individual anonymous information of primary care and secondary care (hospital data from the French Hospital Discharge database [PMSI]), and it covers currently 98.8% of the country population. The SNIIRAM database contains a large number of variables, as previously described.^15^

The access to the SNIIRAM is regulated and requires approval from the “*Institut des Données de Santé*” (IDS, Institute of health data) and the “*Commission Nationale Informatique et Libertés*” (CNIL, the French data protection commission).

#### 3.1.2 Study population

Patients will be included in the study if they are covered by the French national health insurance general scheme, with at least one reimbursement of anticoagulant treatment initiated with an index anticoagulant treatment (either naïve or experienced patients), aged 18 or older at their anticoagulant initiation, and diagnosed with AF in the 24 months prior the anticoagulant treatment initiation.

Patients will be excluded of the study cohorts if they had different types of anticoagulant treatment at the index date, diagnosed with a valvular condition in the 24 months before their anticoagulant initiation or treated for an indication other than stroke prevention in AF in the 6 weeks before their first anticoagulant reimbursement (including index date).

**Figure 2:**
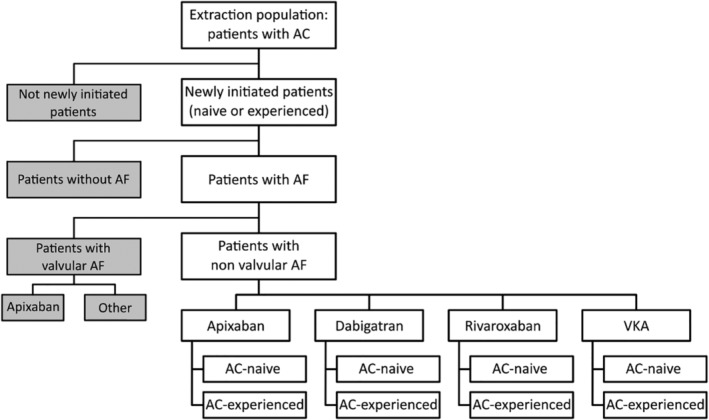
Global flow chart of the study population. AC, anticoagulant; AF, atrial fibrillation; VKA, vitamin‐K antagonist

The study population will be identified in the database through consecutive steps as indicated in Figure 2 and detailed in Supporting Information Appendix 2.

Anticoagulant‐naive patients will be defined as patients with no dispensation of anticoagulant treatment during the 24 months before the index date, and anticoagulant‐experienced patients corresponding to the others. Patients in the experienced group will only be included at their first entry. Therefore, all patients aged ≥18 years and covered by the French national health insurance general scheme, with at least one reimbursement for OAC treatments (VKAs, apixaban, rivaroxaban, or dabigatran) between January 2014 and December 2016, and without use of the same OAC in the 24 months before the index date (ie, date of the first dispensation) will be included. Only the first dispensation meeting this criterion determined the index date, that is, a patient could be included only once in the study.

Then, patients selected were allocated to four distinct sub‐cohorts of OAC‐naive patients (ie, with no dispensation of any OAC during the 24 months before the index date), receiving either VKAs, apixaban, rivaroxaban, or dabigatran during the study period. Anticoagulant‐experienced patients corresponded to the others.

### 3.2 Study outcomes

Primary outcomes:

- *Primary effectiveness outcome* will be the risk of stroke or systemic thromboembolic events which includes ischemic stroke, hemorrhagic stroke, and systemic thromboembolic events.
- *Primary safety outcome* will be the risk of major bleeding which includes intracranial bleeding, gastric duodenal and rectum bleedings, acute posthemmorrhagic anemia, intraocular bleeding, otorrhagia, pericardic bleeding, respiratory bleeding, hemoperitoneum, intra articular bleeding, uterine and vaginal bleeding, and other major bleedings.

Variables used to capture effectiveness and safety outcomes are summarized in Table 1.

**Table 1:** Variables used for effectiveness and safety outcomes

Effectiveness outcome (risk of stroke and systemic thromboembolic events)	Safety outcome (risk of major bleeding)
**Identification of events (ICD‐10 codes collected in PMSI)** **– Ischemic stroke or not specified** Cerebral infarction (except cerebral infarction due to cerebral venous thrombosis, nonpyogenic): *code I63(except I636)* Stroke, not specified as hemorrhage or infarction: *code I64* **– Hemorrhagic stroke** Subarachnoid hemorrhage: *code I60* Intracerebral hemorrhage: *code I61* Other nontraumatic intracranial hemorrhage: *code I62* **– Systemic thromboembolic events** Arterial embolism and thrombosis: *code I74*	**Identification of events (ICD‐10 codes collected in PMSI)** **– Intracranial bleeding** Intracranial hemorrhage: *codes I60 to I62* Epidural hemorrhage: *code S064* Traumatic subdural hemorrhage: *code S065* Traumatic subarachnoid hemorrhage: *code S066* **– Gastric duodenal and rectum bleeding** Oesophageal varices with bleeding: *code I850* Gastro‐oesophageal laceration‐hemorrhage syndrome: *code K226* Gastric ulcer/duodenal ulcer/peptic ulcer/gastrojejunal ulcer with hemorrhage: *codes K250, K252, K254, K256, K260, K262, K264, K266, K270, K272, K274, K276, K280, K282, K284, K286* Acute hemorrhagic gastritis: *code K290* Hemorrhage of anus and rectum: *code K625* Hematemesis: *code K920* Melaena: *code K921* Gastrointestinal hemorrhage, unspecified: *code K922* **– Acute posthemorrhagic anemia**: ***code D62*** **– Intraocular bleeding** Retinal hemorrhage: *code H356* Vitreous hemorrhage: *code H431* Vitreous hemorrhage in diseases classified elsewhere: *code H450* **– Otorrhagia**: ***code H922*** **– Pericardic** Hemopericardium, not elsewhere classified: *code I312* **– Respiratory bleeding** Hemothorax: *code J942* Hemorrhage from respiratory passages: *R04* **– Hemoperitoneum**: ***code K661*** **– Intra articular bleeding** Hemarthrosis: *code M250* **– Uterine and vaginal bleeding** Recurrent and persistent hematuria: *code N02* Other specified abnormal uterine and vaginal bleeding: *code N938* Abnormal uterine and vaginal bleeding, unspecified: *code N939* Postmenopausal bleeding: *code N950* Unspecified hematuria: *code R31* **– Other bleeding** Hemorrhage, not elsewhere classified: code *R58* Traumatic secondary and recurrent hemorrhage: code *T792*

Abbreviations: ICD‐10, International Classification of Diseases, 10th Revision; PMSI, French Hospital Discharge database.

Secondary outcomes:

- Risk of occurrence of a composite morbidity criterion: unadjusted incidence rate in each sub‐cohort, composite morbidity criterion being defined by stroke, systemic thromboembolic events, and/or major bleeding, whichever occurs first.
- Risk of all‐cause mortality: unadjusted incidence rate of all‐cause death in each sub‐cohort.
- Major characteristics of patients and comorbidities, which are summarized in Table 2.
- Treatment patterns at anticoagulant initiation: Type of the prescriber initiating the anticoagulant treatment (general practitioners, office‐based cardiologists, hospital‐based physicians, and others), dispensed dosages, number of dispensed packaging at anticoagulant initiation, co‐prescription of dispensed treatments (other anticoagulants, antiplatelet agents, nonsteroidal anti‐inflammatory drugs, serotonin‐specific reuptake inhibitors, strong inhibitors of both CYP3A4 and P‐gp, HIV protease inhibitors, anticonvulsants, strong inducer of hepatic enzymes, and antiarrhythmic drugs) in each sub‐cohort.
- Treatment patterns over time:
  - Adherence to treatment: Continuous Measure of Medication Acquisition version 7 (CMA 7).^16^
  - Persistence: number and percentage of persistent and non‐persistent patients over the 6 months and the 12 months after index date, time‐to‐event of persistence, median duration of persistence, number and percentage of non‐persistent patients with other anticoagulant therapy administered after discontinuation, during the gap, the number and percentage of persistent patients who had concomitant dispensings of other anticoagulant treatment over the exposure period of the studied anticoagulant, the number and percentage of non‐persistent patients who switched from studied anticoagulant treatment and re‐exposed after the switch during a 1‐year period, in each sub‐cohort.
- Healthcare resources utilization description: use of medical visits, nurse acts, drugs packages delivered, biology acts, medical procedures, hospital stays, and sick leaves will be reported as:
  - The number and percentage of patients with at least one reimbursement of a such healthcare resource use during the follow‐up period.
  - The mean, SD, median, percentiles 10 and 90 of the number of care, only in patients with at least one reimbursement of the healthcare during the follow‐up period.

**Table 2:** Major patients characteristics and comorbidities evaluated for each anticoagulant

Sociodemographic characteristics: median age, sex ratio, region of residence, free‐access‐to‐care status
AF characteristics: time since AF diagnosis
LTD status distribution (LTD type, ICD‐10 code for diagnosis)
Previous hospital stay: number and total length of hospital stays
Previous exposure to anticoagulant treatment (class, molecule) over the three previous years, for anticoagulant‐experienced patients
Thromboembolism risk factors: CHADS2 mean score, CHA2DS2 VASc mean score, and distribution according to the scores
Bleeding risk factors: HASBLED mean score and distribution according to the score
Charlson mean score and distribution according to the score

Abbreviations: AF, atrial fibrillation; ICD‐10, International Classification of Diseases, 10th Revision; LTD, long‐term disease.

The study is registered at ClinicalTrials.gov, number NCT02640222. No other substudies have yet been planned.

### 3.3 Ethics and confidentiality of study data

This study does not require review and approval by ethics committees or informed consent. The confidentiality of records that could identify patients within the database will be protected, respecting the privacy and confidentiality rules in accordance with the applicable regulatory requirements. This database analysis will use anonymous patient's data.

### 3.4 Statistical analyses

#### 3.4.1 Primary effectiveness and safety outcomes

For primary effectiveness outcome, estimates by anticoagulant treatment and for both populations (anticoagulant‐naive and anticoagulant‐experienced patients) of:

- The number and percentage of patients presenting at least a stroke and/or a systemic embolic event during the 12 months after index date, and during the overall follow‐up period. This description will be also conducted by diagnoses sub‐division presented in Table 1.
- The unadjusted incidence rate: number of first event of stroke and/or systemic embolism by 100 person‐years and during the overall follow‐up period will be estimated by anticoagulant treatment. Person‐years are defined with the sum of follow‐up durations of the at‐risk population. The corresponding two‐sided 95% CI will also be represented using Poisson distribution.
- The time‐to‐event of first occurrence of stroke and/or systemic thromboembolism will be estimated and plotted using cumulative incidence function (the cumulative probability of failure from a specific cause over time) accounting for differences in follow‐up time and the competing risk of mortality (if mortality is greater than 10%). In the presence of competing risks of mortality, informative censoring of the observation time at death will be used, as mortality will be considered a competing risk for observing stroke and systemic thromboembolic events since patients who die before an event is observed cannot go on to have an event. In the absence of competing risks, the differences in follow‐up time will be accounted for through non‐informative censoring of the observation time at the end of follow‐up for each patient. Overall, the causes of censoring of each treatment group will be reported once the results will become available. Cumulative incidence curves will present the event risk over time (within first 12 months) for each newly initiated anticoagulant treatment while accounting for the competing risk of mortality (if needed) and differences in follow‐up time.
- Median duration to occurrence of first event of stroke and/or systemic thromboembolism: median duration (in days) between the index date and the date of the first studied event will be computed for patients with the event.

For primary safety endpoint, estimates by anticoagulant treatment and for both populations (anticoagulant‐naive and anticoagulant‐experienced patients) will be addressed by using the same methods as described for the primary effectiveness endpoint.

In order to account for confounding in the comparative analyses, several methods will be performed to compare groups of patients with the similar characteristics including: adjustment on propensity score (estimation of the average treatment effect in the entire population if the treatment might be offered to every member of the population, ATE), matching on propensity score (estimation of the average treatment effect of matched subjects who ultimately received the treatment, ATT), and two other comparative approaches as sensitivity analyses (using high dimensional propensity scores, and adjustment on confounders).

The main method will consist in propensity score adjustment to compare groups of patients with the same characteristics. The propensity score is the probability of treatment assignment conditional on observed baseline characteristics. Propensity score is used when selection bias due to non‐random treatment assignment is likely, because of observational data. Three different propensity scores will be performed according to the comparison (apixaban vs dabigatran, apixaban vs rivaroxaban or apixaban vs VKAs). The propensity score conventional method will be used, which is to derive a score based on all the confounding factors previously identified. The probability to be treated with a given therapeutic combination will be estimated using a logistic regression model (with all confounding factors and treatment as dependent variable). Several checks will be performed to ensure a good balance of propensity score and of covariates between apixaban and comparison groups:

- First, the treatment group propensity score distribution will be analyzed with a graphical representation. If no overlap between distributions is observed, patients will be defined as different, so comparisons would not be performed.
- Then, the balance of propensity score across treatment and comparison groups will be checked, with comparison of means. The balance of covariates across treatment and comparison groups will be checked using standardized difference.^17^

If no balance, a new propensity score model will be specified (deletion of variables or transforms).^17^

#### 3.4.2 Secondary outcomes

For the secondary outcomes, the comparison between patients initiating apixaban and patients initiating each of the other anticoagulant treatments (dabigatran, rivaroxaban, and VKAs) will be performed only in the four sub‐cohorts of anticoagulant‐naive patients.

For all other descriptive objectives, the analyses will be performed in the eight sub‐cohorts.

Estimation by anticoagulant treatment and for both populations (anticoagulant‐naive and anticoagulant‐experienced patients) as follows:

- Quantitative variables will be described with the sample size, mean, standard deviation, median, interquartile range, minimum and maximum.
- Qualitative and ordinal variables will be described with the sample size and frequencies.

Analyses in anticoagulant‐naive and anticoagulant‐experienced populations as follows:

- The major characteristics of patients will be described for each anticoagulant treatment.
- Treatment patterns at anticoagulant initiation, over time, and concomitant treatment will be tabulated by anticoagulant treatment.
- Time‐to‐discontinuation will be estimated and plotted using cumulative incidence function by anticoagulant treatment.
- Healthcare resources utilization will be described by anticoagulant treatment.

For each endpoint of interest in the study, only the first evidence of its event will be considered in the analyses. Cox models have been chosen to align with the methodology used in other OAC publications. As this study has been conducted to address a specific request from French health authorities, the comparability of the methods with other OAC publication was an important contributor of the analytical choices made a priori.

Cox models or Fine Gray models are planned to be used in the analyses. The final choice of model will be based on the observed death rates, that is, in case of a high likelihood of competing risk of death, Fine Gray models will be used rather than Cox models.

## 4 DISCUSSION

Registration clinical trials, such as RE‐LY,^4^ ROCKET‐AF,^5^ or ARISTOTLE^6^ are required to mandate strict inclusion and exclusion criteria and to enforce tight control of both intervention therapy and comparator. These trials established the effectiveness and safety of NOACs in the selected populations from randomized trials. Nevertheless, non‐interventional studies and registries allow to confirm the effectiveness and safety of newly approved medications across a wider range of patients (characteristics, comorbidities, healthcare access, disease history, etc.) treated in routine clinical practice, an environment in which treatment control is not as strict as in randomized clinical trials.^18^, ^19^

Recent, non‐interventional studies and registries on NOACs have confirmed the results of randomized clinical trials.^9^, ^10^, ^11^, ^12^, ^13^ Nevertheless, while it is reassuring that the body of emerging clinical practice data on NOACs in other countries largely mirrors that in clinical trials,^9^, ^10^, ^11^, ^12^, ^13^ only little data is available for France. To the best of our knowledge, the NAXOS study will be the first nationwide clinical practice study in France that will evaluate all available NOACs (dabigatran, rivaroxaban, and apixaban) effectiveness and safety. It will also provide additional data regarding patients and disease characteristics, comorbidities and treatment history in NVAF patients initiating a new anticoagulant treatment and allow the comparison of these characteristics between patients treated with apixaban and with other anticoagulants. Moreover, in addition to confirm effectiveness and safety outcomes of clinical trials, clinical practice data can also give information on medication prescription in a clinical practice setting. A recent observational propensity‐weighted nationwide cohort study in Denmark,^20^ which involved 61 678 patients with AF who were naive to oral anticoagulants, showed that patients receiving VKAs or NOACs tend to differ in their characteristics and comorbidities. Among the studied population, 57% received warfarin, 21% dabigatran 150 mg, 20% rivaroxaban 20 mg, and 10% apixaban 5 mg. Patients receiving apixaban or rivaroxaban had more frequent history of previous stroke, systemic embolism, vascular disease and bleeding, while patients receiving dabigatran were younger and less renal impaired warfarin‐treated patients had more frequently a history of vascular disease, hypertension, renal impairment, chronic obstructive pulmonary disease and cancer. The NAXOS study will provide insights on these characteristic differences among different anticoagulants, among anticoagulant‐experienced patients. Indeed, other registries studies noted that patient who initiate treatment with warfarin have a higher bleeding risk when compared to patients receiving dabigatran, warfarin experienced switchers, or patients remaining on warfarin.^21^ The present study will also allow to evaluate this hypothesis in a clinical practice setting with respect to patients initiating apixaban. In addition, this analysis is complementary to the PAROS (Apixaban in the prevention of stroke and systemic embolism in patients with non‐valvular atrial fibrillation in France) study^22^ which will compare the characteristics of patients with AF newly anticoagulated with either VKAs or NOACs.

Despite the numerous advantages related to the use of the SNIRAM database in pharmacoepidemiology studies, this database has limitations. First, there is a risk of incomplete data collection due to the research method using identification of patients through ICD‐10 diagnosis in the SNIIRAM database. Therefore, an algorithm needs to be applied to catch AF patients and can induce a diagnosis bias. Second, only major bleeding resulting in hospitalization is collected through PMSI. Less severe bleeding, requiring emergency care but no hospitalization will not be identified in the database. Third, due to the claim‐based retrospective nature of our study, treatment discontinuation of VKA could be biased due to the nature of individual variable dosing of VKA. Nevertheless, algorithms were performed to overcome these issues. In addition, as we used INR testing during hospitalization as a proxy for VKA dispensation, we might have miss patients who presented temporary interruption of their anticoagulant treatment. As the HAS requested clinical practice data documenting the therapeutic benefit of apixaban under actual conditions of use, compared with the standard oral anticoagulant treatment recommended in France (vitamin‐K antagonists, dabigatran, and rivaroxaban), only apixaban off‐label use were collected and therefore, off‐label data on other anticoagulants could not be reported. Fourth, one of the main limitations of this database, as is the case for many administrative databases, is the lack of information for certain clinical or biological risk factors for stroke, thromboembolism or bleeding. Without such information it is not possible to compare the distribution of these risk factors between anticoagulant treatments. Adjusting/matching for large numbers of covariates ascertain from patients' healthcare claims data improved control of confounding, as these variables could collectively be proxies for unobserved factors. Finally, future analyses of this study could also include complementary comparative approaches, such as using IPTW, which is another and probably better method to estimate an average treatment effect in the entire population if the treatment might be offered to every member of the population (ATE).

## 5 CONCLUSION

The NAXOS study will allow the generation of new data regarding the characteristics and management of NVAF patients and unique data on the effectiveness and the safety of NOACs in a clinical practice setting in France.

## CONFLICT OF INTEREST

Fabien Picard: Speaker's and/or consulting fees: Biotronik and Sanofi. Gregory Ducrocq: Speaker's and/or consulting fees: AstraZeneca, Biotronik, BMS, Daiichi Sankyo, Sanofi; CEC: Sanofi, Philips; DSMB: Abbot, MicroPort; Travel Fees: AstraZeneca, Biotronik. Nicolas Danchin: research grants: Amgen, AstraZeneca, Bayer, BMS, Boehringer‐Ingelheim, Daiichi Sankyo, Eli‐Lilly, MSD, Pfizer, Sanofi; advisory panels or lecture fees: Amgen, AstraZeneca, Bayer, BMS, Boehringer‐Ingelheim, Bouchara‐Ricordati, Daiichi Sankyo, Eli‐Lilly, MSD, Novo‐Nordisk, Servier, Sanofi. Bruno Falissard: Consultant for E. Lilly, BMS, Servier, SANOFI, GSK, HRA, Roche, Boeringer Ingelheim, Bayer, Almirall, Allergan, Stallergene, Genzyme, Pierre Fabre, AstraZeneca, Novartis, Janssen, Astellas, Biotronik, Daiichi Sankyo, Gilead, MSD, Lundbeck, Stallergene, Actelion, UCB, Otsuka, Grunenthal, ViiV. Olivier Hanon: Advisory panels or lecture fees: BMS, Pfizer, Daichi Sankyo, Boehringer‐Ingelheim, Bayer, Novartis, Sanofi‐Aventis, AstraZeneca, Servier, Vifor. Isabelle Mahé: Research grants from BMS‐Pfizer, Leo Pharma, Speaker and consulting Fees: BMS‐Pfizer, Leo Pharma, Bayer. Philippe Gabriel Steg: Research grant from Merck, Sanofi, and Servier; speaking or consulting fees from Amarin, Amgen, AstraZeneca, Bayer, Boehringer‐Ingelheim, Bristol‐Myers‐Squibb, CSL‐Behring, Janssen, Lilly, Merck, Novartis, Pfizer, Regeneron, Sanofi, Servier. Eric Van Ganse: Grants and personal fees from ALK ABELLO, Bayer, BMS, GlaxoSmithKline, Merck Sharp and Dohme, personal fees from PELyon. Marine Ginoux: Employee of PELyon. Manon Belhassen: Employee of PELyon. Cinira Lefevre: Employee of BMS. François‐Emery Cotte: Employee of BMS. The authors declare no potential conflict of interests.

## Supporting information


**Appendix 1:** Study organization.

**Appendix 2:** Criteria and algorithm used to build study population.
