# Supplementary material for: EvaluatioN of ApiXaban in strOke and systemic embolism prevention in patients with non‐valvular atrial fibrillation in clinical practice Setting in France, rationale and design of the NAXOS: SNIIRAM study
Source: Clin Cardiol. 2019 Jul 17;42(10):851–9. doi: 10.1002/clc.23231 (PMC6788467; doi:10.1002/clc.23231)
Supplement: Supplementary file 1 — Appendix 1: Study organization. [file CLC-42-851-s001.docx]

**Supplementary Appendix 1 : Study Organization**

**Project team**

*Study Directors*

Dr Claire Chartier ; Bristol-Myers Squibb ; 3 rue Joseph Monier 92500 Rueil-Malmaison France

Virginie Vannier-Moreau ; Bristol-Myers Squibb ; 3 rue Joseph Monier 92500 Rueil-Malmaison France

*Project Leads*

Pierre Marie ; Bristol-Myers Squibb ; 3 rue Joseph Monier 92500 Rueil-Malmaison France

*Biostatisticians*

Benoit Caritey ; Bristol-Myers Squibb ; 3 rue Joseph Monier 92500 Rueil-Malmaison France

Anne Filipovics ; Bristol-Myers Squibb ; 3 rue Joseph Monier 92500 Rueil-Malmaison France

*Advisors*

HEOR study expert advisor: François-Emery Cotte ; ; Bristol-Myers Squibb ; 3 rue Joseph Monier 92500 Rueil-Malmaison France

CORDS Research: advisor Cinira Lefevre ; Bristol-Myers Squibb ; 3 rue Joseph Monier 92500 Rueil-Malmaison France

**Coordinating center : PELyon**

*Study Directors*

Dr Eric Van Ganse ; PELyon; Faculté d’Odontologie ; 11 rue Guillaume Paradin 69008 Lyon

Dr Manon Belhassen ; PELyon; Faculté d’Odontologie ; 11 rue Guillaume Paradin 69008 Lyon

*Project Leads*

Faustine Valentini ; PELyon; Faculté d’Odontologie ; 11 rue Guillaume Paradin 69008 Lyon

*Biostatisticians*

Marine Ginoux ; PELyon; Faculté d’Odontologie ; 11 rue Guillaume Paradin 69008 Lyon

Maëva Nolin ; PELyon; Faculté d’Odontologie ; 11 rue Guillaume Paradin 69008 Lyon

Flore Jacoud ; PELyon; Faculté d’Odontologie ; 11 rue Guillaume Paradin 69008 Lyon

**Scientific board**

Pr Nicolas Danchin; Cardiologist ; Hôpital Européen Georges Pompidou Service de Cardiologie ; 20 rue Leblanc
75015 Paris

Pr Bruno Falissard; Epidemiologist/Methodologist; INSERM U669 Maison de Solenn ; 97 Bld de Port Royal 75679 Paris cedex 14

Pr Olivier Hanon; Geriatrician/Gerontologist; Hôpital Broca Service de gériatrie 1 54-56 rue Pascal 75013 Paris

Pr Isabelle Mahe; Internist; Hôpital Louis Mourier Service de Médecine Interne Université Paris 7, APHP 178 rue des Renouillers 92700 Colombes

Pr Philippe Gabriel Steg; Cardiologist; Hôpital Bichat Département de Cardiologie 46 rue Henri Huchard 75018 Paris

Pr Emmanuel Touze Neurologue; CHU Côte de Nacre Service de Neurologie Avenue de la Côte de Nacre 14000 Caen »
