# Supplementary material for: EvaluatioN of ApiXaban in strOke and systemic embolism prevention in patients with non‐valvular atrial fibrillation in clinical practice Setting in France, rationale and design of the NAXOS: SNIIRAM study
Source: Clin Cardiol. 2019 Jul 17;42(10):851–9. doi: 10.1002/clc.23231 (PMC6788467; doi:10.1002/clc.23231)
Supplement: Supplementary file 2 — Appendix 2: Criteria and algorithm used to build study population. [file CLC-42-851-s002.docx]

**Supplemental Appendix 2: Criteria and algorithm used to build study population**

1. **Inclusion and exclusion criteria**

Inclusion Criteria

Patients will be included in the study if they met all the following inclusion criteria during the study period:

- Patient covered by the French national health insurance general scheme,
- Patients with at least one reimbursement of anticoagulant treatment (acenocoumarol, warfarine or fluidione for VKA treatments, apixaban, dabigatran or rivaroxaban for NOACs),
- Patients initiated with an index anticoagulant treatment (either naïve or experienced patients),
- Patients aged 18 or older at their anticoagulant initiation,
- Patient diagnosed with AF in the 24 months prior the anticoagulant treatment initiation.

Exclusion Criteria

Patients will be excluded of the study cohorts if they met the following criteria:

- Patients with different types of anticoagulant treatment at the index date,
- Patients diagnosed with a valvular condition in the 24 months before their anticoagulant initiation

Patients in the anticoagulant-naive cohorts will be excluded for all objectives (except for the description of demographic and clinical characteristics), if they meet the following criterion:

- Patients possibly treated for an indication other than stroke prevention in AF in the 6 weeks before their first anticoagulant reimbursement (including index date).

1. **Algorithm used to identify study population in the database**

The study population was identified in the database through consecutive steps as indicated in the Figure 2: 1) Identification of patients with anticoagulant treatment. 2) Identification of patients newly initiating the index anticoagulant treatment. 3) Identification of adult patients. 4) Identification of AF patients 5) Identification of NVAF patients. 6) Identification of four cohorts according to the first anticoagulant treatment newly initiated. 7) Identification of anticoagulant-naive or anticoagulant-experienced sub-cohorts. 8) Identification of patients possibly treated for indication other than stroke prevention in AF, in the anticoagulant-naive cohorts only. In final, 8 sub-cohorts were obtained.

**Algorithm for AF patient identification**

AF patients could not be identified in SNIIRAM through simple diagnosis information. The identification of AF patients is based on four different indicators, including confirmed and probable cases of AF:

I. Confirmed AF:

1) Diagnoses identified through Long Term Disease (LTD) in the 24 months before and the 30 days after index date, entered in SNIIRAM database by physicians. AF is included in the Fifth LTD, covering ‘Severe cardiac insufficiency, severe valvular and congenital heart diseases and severe cardiac rhythm disorders.’ The ICD-10 code I48 (Atrial Fibrillation) associated to LTD will flag an AF patient.

2) Main or associated diagnoses of hospitalizations in the 24 months before index date: each hospitalization with a main or associated diagnoses coded ‘Atrial fibrillation and flutter’ (ICD-10 code I48) will flag an AF patient.

II. Probable AF:

3) Drug markers according to medical experts: any anti-arrhythmic drugs dispensed concomitantly (i.e. dispensed between the six weeks before and the 15 days after index date) with VKAs or NOACs in the database flagged an AF patient. Anti-arrhythmic treatments taken into account will be the specialties of the therapeutic class C01B, i.e. specialties with one of the following active molecule: flecainide, amiodarone, propafenone, disopyramide, cibenzoline, hydroquinidine, dronedarone, verapamil, diltiazem, or digoxin.

4) Medical markers according to medical experts: any cardioversion or ablation in the 24 months before index date flagged an AF patient.

Patients presenting at least one of these four indicators (LTD, hospitalization, drug or medical markers) were considered as treated for an indication of AF.

**Algorithm to exclude valvular-AF patients**

After the identification of AF patients, a further process was undertaken to exclude those which had a valvular AF according to the ESC definition[3]: the term valvular AF is used to imply that AF is related to rheumatic valvular disease (predominantly mitral stenosis) or prosthetic heart valves.

- Patients having had a valvular replacement in their medical history will be excluded from NVAF patient population. These patients will be flagged through medical procedures in the 24 months before index date.

- Patients with a diagnosis of rheumatic cardiopathy and valvular disorders, identified through LTD in the 24 months before and the 30 days after index date, will be excluded from NVAF patient population.

- Patients with a main or associated diagnosis of rheumatic cardiopathy and valvular disorders, for hospitalizations in the 24 months before index date, will be excluded from NVAF patient population.

**Algorithm to exclude non AF anticoagulant treatment**

In addition, a further process for identification of patients treated with anticoagulant for other indications than AF is based on different indicators, calculated in the 6 weeks before index date (including index date):

- Patients with a diagnosis of venous thromboembolism or pulmonary embolism

- Patients with a main or associated diagnosis of venous thromboembolism or pulmonary embolism for hospitalizations.

- Patients with a hospital discharge for a total hip replacement, a total knee replacement or an orthopaedic surgery.

Anticoagulant-naive patients presenting at least one of these indicators will be excluded.

Patients not presenting at least one of these three indicators will be considered as treated for an indication of NVAF. Patients with at least one of these three indicators and newly treated with apixaban will be defined as off-label use patients.
